# Cannabidiol enhances cerebral glucose utilization and ameliorates psychopathology and cognition: A case report in a clinically high-risk mental state

**DOI:** 10.3389/fpsyt.2023.1088459

**Published:** 2023-03-03

**Authors:** Dagmar Koethe, Cathrin Rohleder, Lutz Kracht, F. Markus Leweke

**Affiliations:** ^1^Brain and Mind Centre, Faculty of Medicine and Health, The University of Sydney, Sydney, NSW, Australia; ^2^Department of Psychiatry and Psychotherapy, Central Institute of Mental Health, Medical Faculty Mannheim, Heidelberg University, Mannheim, Germany; ^3^Institute of Radiochemistry and Experimental Molecular Imaging, Faculty of Medicine and University Hospital Cologne, University of Cologne, Cologne, Germany; ^4^Endosane Pharmaceuticals GmbH, Berlin, Germany; ^5^Max-Planck-Institute for Metabolism Research, Cologne, Germany

**Keywords:** CBD, cannabinoids, clinical high at-risk mental state (CHARMS), at-risk mental state (ARMS), ultra-high risk (UHR) for psychosis, FDG-PET (F-18-fluorodeoxyglucose-positron emission tomography), Cognitive function, psychopathological symptoms

## Abstract

Adolescent individuals often present with subtle, sub-threshold psychiatric syndromes that fluctuate or persist for years. These symptoms have been classified as Clinically High-Risk mental states (CHR), negatively affecting these individuals’ psychosocial development and integration by reducing performance and affecting interpersonal relations. The pathophysiological underpinnings have not been studied in detail, contributing to the current lack of appropriate intervention strategies. This case report sheds new light on potential pathophysiological mechanisms of this condition, which may be addressed by novel treatment approaches such as cannabidiol. A 19-year-old student presented to our early intervention center with a marked cognitive decline within 6 months, anhedonia, ambivalence, social withdrawal, poverty of speech, and brief intermittent psychotic symptoms (delusions and hallucinations). He was diagnosed with CHR state, and we decided to treat him with the non-psychotomimetic phytocannabinoid cannabidiol. Cannabidiol is a promising compound carrying an orphan drug approval for rare certain childhood epilepsy types and is under investigation as an antipsychotic compound with a new mechanism of action compared to existing antipsychotics. We investigated the effect of oral cannabidiol (600  mg per day) over 4 weeks on psychopathology and cerebral glucose utilization. We observed no relevant side effects but a significant clinical improvement. In addition, positron emission tomography (PET) showed a considerable increase in cerebral [^18^F]fluoro-2-deoxyglucose (FDG) uptake in various brain regions.

This finding suggests that cannabidiol may enhance cerebral glucose utilization, possibly *via* activation of peroxisome proliferator-activated receptor-gamma (PPAR-γ) by its endogenous ligand anandamide or related N-acylethanolamines. This mechanism may represent a new innovative treatment approach for CHR, especially given that many individuals with CHR and early psychosis do not substantially benefit from current psychopharmacological interventions.

## 1. Introduction

Individuals in a Clinical-High-Risk mental state (CHR) show mild attenuated psychotic symptoms (APS) or brief, limited intermittent psychotic symptoms (BLIPS), as well as more general and unspecific psychiatric symptoms. Moreover, CHR is often accompanied by neurocognitive and functional impairments (1, 2). These symptoms often fluctuate or persist for years and affect the psychosocial development of these individuals. In particular, the impairments in social and role functioning and reduced neurocognitive performance are critical, as these are associated with poor long-term clinical outcomes and a higher conversion rate to psychosis (1–4). While these individuals are often help-seeking and various pharmacological interventions such as antipsychotics or antidepressants are used off-label, no pharmacological treatment is approved because the condition has long been understood as an at-risk state of psychosis rather than a disorder on its own. However, clinical care for individuals with persistent sub-threshold psychotic symptoms is undoubtedly indicated, given the functional impairment in this population (5).

The phytocannabinoid cannabidiol is a promising novel therapeutic compound for treating psychosis (6, 7). In early psychosis patients, cannabidiol had a superior side-effect profile and efficacy similar to amisulpride – a highly effective second-generation antipsychotic (8). Due to its excellent tolerability and promising efficacy regarding positive and particular negative symptoms and its innovative new mechanisms of action (6–10), we decided to offer a respective treatment with cannabidiol to an adolescent CHR patient, which the ethics committee of the University of Cologne accepted. Here we report the effects of a 30-day treatment on psychopathology, cognition, and cerebral glucose utilization.

## 2. Case description

A 19-year-old Caucasian male student presented with a marked cognitive decline within 6 months, anhedonia, ambivalence, social withdrawal, poverty of speech, and BLIPS, particularly delusions and hallucinations. After an initial psychological assessment at the Cologne Early Recognition and Intervention Center, he was classified as a CHR individual, and further examinations to clarify the initial diagnosis were recommended. Two months later, he presented to the Department of Psychiatry and Psychotherapy, University of Cologne, for more detailed examinations. The results of the clinical and neurological examination and investigations, including brain magnetic resonance imaging (MRI), electroencephalogram (EEG), and extensive testing of blood and cerebrospinal fluid (CSF), were within the normal range. Besides an uncle with bipolar disorder, he had no family history of other psychiatric or neurological diseases. He had no psychiatric history, was antipsychotic-naïve, and had not received psychological treatment. Cannabis was self-reporting as the only illicit drug consumed four times in his lifetime at the age of 15 years in social contexts in minor to moderate doses; urine drug screening was negative. The initial CHR diagnosis was confirmed based on these findings, and treatment options were discussed.

**Figure 1:**
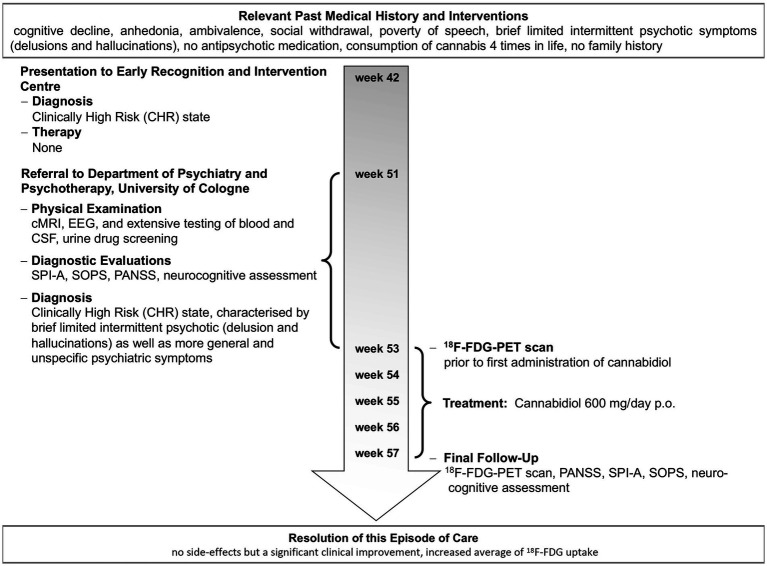
Timeline of important events and examinations in the patient’s history and individual treatment.

After written informed consent of the patient and a written report to the local ethics committee of the University of Cologne for administering oral, active pharmaceutical ingredient (API)-grade cannabidiol (THC Pharm, Frankfurt, Germany) capsules for an individual treatment attempt, he was treated with pure cannabidiol 600 mg/day orally for 30 days under close clinical monitoring. He received no other additional drug or psychological treatments during that time. Neuropsychological testing and [^18^F]fluoro-2-deoxyglucose (^18^F-FDG) positron emission tomography (PET) were performed prior to and post-treatment (Figure 1). Psychopathology was assessed prior to and post-intervention using the Positive and Negative Symptoms Scale (PANSS), the Schizophrenia Proneness Instrument—Adult version (SPI-A), and the Structured Interview for Prodromal Syndromes (SIPS). The neuropsychological assessment included the following tests: Auditory Verbal Learning Test (AVLT; immediate memory, delayed recall, and recognition memory), Verbal Fluency Test (VF; verbal functioning), Letter Number Sequencing (Digit Span Test; verbal working memory), Spatial Working Memory Test (SWM), Trail-Making Test (TMT; visual attention, cognitive flexibility (task switching), and visuomotor speed), Continuous Performance Test (CPT; sustained attention and concentration), and Visual Backward Masking Task (VBM; visual processing of a target stimulus).

**Figure 2:**
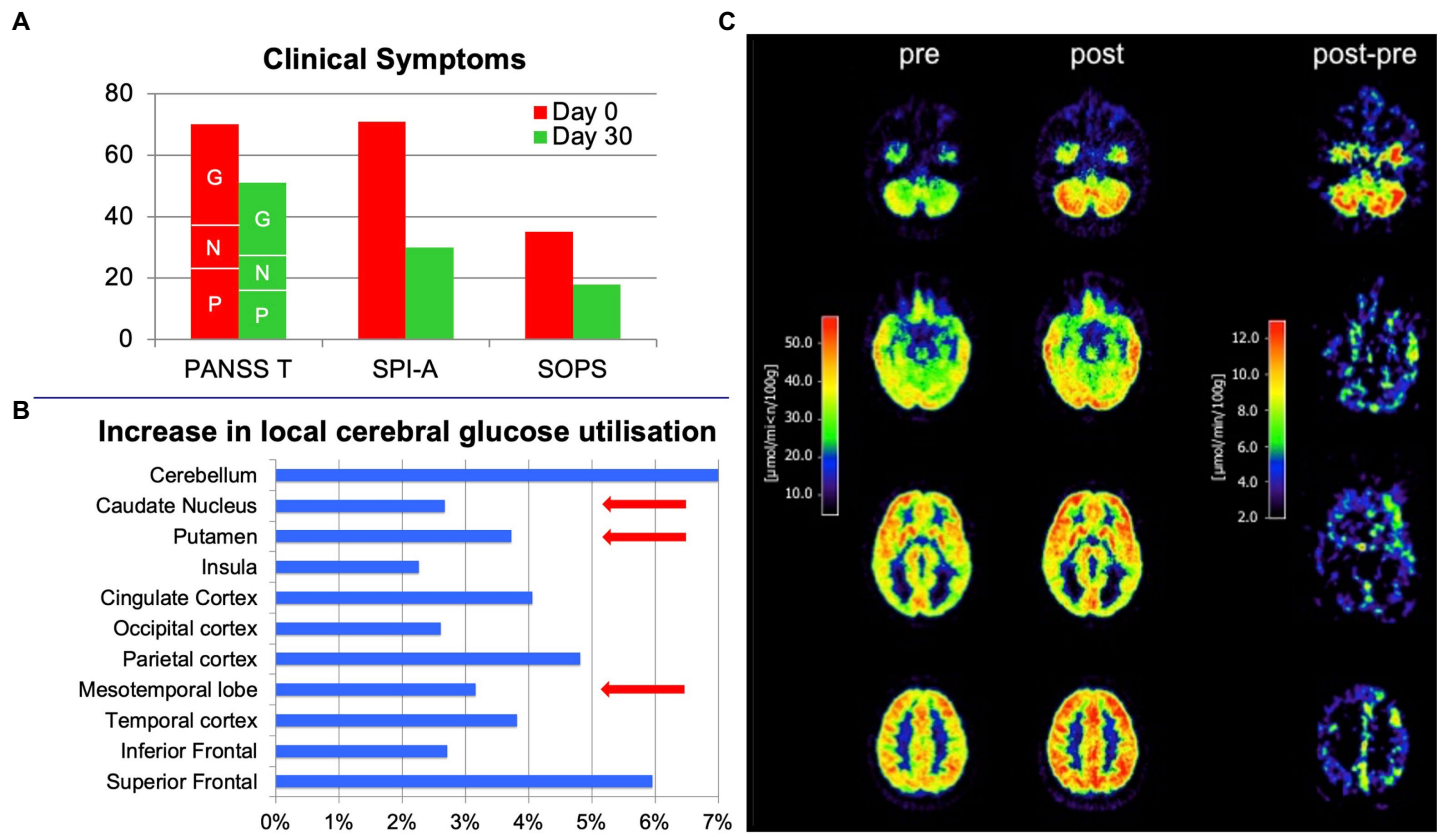
Effects of treatment with 600 mg oral cannabidiol per day in a 19-year-old male with clinically high mental risk before (pre) and on day 30 (post). **(A)** Positive and Negative Symptoms Scale (PANSS; P: Positive, N: Negative, G: General subscore segments marked by respective letters), the Schizophrenia Proneness Instrument - Adult (SPI-A) and the Structured Interview for Prodromal Syndromes (SIPS) before (red bars) and after (green bars) treatment. Significant clinical improvement was observed, reflected by the substantial reduction in scale scores. **(B)** FDG positron emission tomography scans of the brain before (pre) and after treatment (post) and the difference between post- and pre-treatment. **(C)** Increase in local cerebral glucose utilization after treatment in specific brain regions (in %). Brain regions (striatum, medial temporal cortex and midbrain) previously reported to be functionally modulated in CHR individuals by a single oral dose of 600 mg cannabidiol in a functional MRI study (11) are highlighted by red arrows.

Medication adherence was notable during the treatment period, and no adverse events or side effects (in particular no extrapyramidal symptoms, no weight gain, no changes in blood pathology, and no sexual dysfunction), but a substantial clinical improvement were observed (Scores on day 0/day 30: PANSS Total 70/51 [PANSS Positive 23/16; PANSS Negative 14/11; PANSS General 33/24]; SPI-A 71/30; SIPS 35/18; Figure 2A), clinically notable already at day seven. Accordingly, neuropsychological testing revealed a marked improvement in cognition – particularly in attention (CPT, TMT), visual processing (VBM), and visuomotor speed (TMT), as well as working memory (SWM). A marked increase in brain glucose metabolism accompanied the improvements in psychopathology and cognition. PET scans were acquired on an ECAT EXACT HR scanner (Siemens-CTI, Knoxville, TN) in 3D mode. 92.5 MBq (2.5 mCi) FDG was administered. Scans between 20 and 60 min of data acquisition and multiple arterialized venous blood samples were used to calculate the metabolic rate of glucose (MRglc) based on the Sokoloff model with the adaption of KI to quantify activity with a lumped constant of 0.52 for a standard human brain.

Initial PET scanning revealed a mildly enhanced FDG uptake in frontal brain areas (analyzed by using the free and open-source software Statistical Parametric Mapping [SPM]), while other brain regions were comparable to scans of healthy controls. The average FDG uptake (standardized uptake value (SUV)) was substantially increased after cannabidiol treatment (Figures 2B,C). At baseline, SUVs ranged from 20.14 to 45.67 μmol/min/100 g and, after 30 days, from 21.47 to 48.63 μmol/min/100 g. The absolute differences ranged from −0.48 (negative difference solely in the right cuneus) to up to +7.43 (left gyrus precentralis; Figures 2B,C).

## 3. Discussion

Consistent with our observation, a relative metabolic increase in unmedicated schizophrenia patients was found in different studies, showing higher glucose utilization in almost all quantified regions compared to healthy volunteers, potentially related to a disruption of cortico-striato-thalamic feedback-loops (12). In contrast, most studies reported lower metabolic rates in fronto-thalamic circuits of treated or chronic schizophrenia patients (13). In CSF, significantly higher glucose levels were found in antipsychotic-naïve first-onset schizophrenia suggesting cerebral alterations in glucoregulatory processes, potentially intrinsic to the disease (14). Furthermore, it was observed that regional brain glucose metabolism changes predict neurocognitive performance in schizophrenia (15). A reduced glucose utilization may be related to a suppression of specific neuronal networks in treated or chronic schizophrenia due to downregulation of the dopaminergic tone. A downregulation of cerebral glucose utilization may therefore reflect a “suppression of mal-function” rather than a “normalisation” of function. The activation of the endocannabinoid system *via* anandamide will likely strengthen the homeostatic effect of this system and thereby “normalize” systemic function and, at the same time, upregulate cerebral glucose utilization (16).

Consistent with this hypothesis, Brett et al. (17) showed region-specific increases in cerebral glucose utilization in the rat brain [prefrontal cortex (namely, ventral and lateral orbital cortex) and ventral pallidum] induced by cannabidiol. Therefore, in line with more recent findings in animals (18), the increased glucose metabolism appears to be due to cannabidiol administration and may be associated with improving psychopathology and neurocognition.

The underlying mechanism might be an increased glucose metabolism *via* activation of the peroxisome proliferator-activated receptor-gamma (PPAR-γ) (19), which plays an essential role in glucose homeostasis and neuroinflammation (20, 21). It has been reported that cannabidiol may bind and activate PPAR-γ directly (22). However, also an indirect PPAR-γ activation through the endogenous PPAR-γ ligands anandamide and related N-acylethanolamines (oleoylethanolamide [OEA] and palmitoylethanolamide [PEA]) (20) may occur, as increased anandamide, OEA, and PEA serum levels were reported to be associated with cannabidiol treatment (8). The direct or indirect activation of PPAR-γ by cannabidiol may represent one of the various other possible mechanisms relevant to the promising antipsychotic effects of cannabidiol (23). This may be particularly interesting in the context of long-standing observations of impaired glucose homeostasis and insulin resistance in general and in the brain in schizophrenia (24).

The strength of our case report lies in the monotherapy with cannabidiol and the consistent and repeated (pre- and post-treatment) clinical and technical assessments of our patient, who did not receive any CHR-related therapy before the individual treatment attempt with cannabidiol. However, the single-case approach is undoubtedly a relevant limitation, and a larger randomized, controlled, double-blind trial is needed to draw consistent conclusions. Due to the unblinded single-case approach, we cannot entirely rule out that the observed symptom improvements and increased FDG-uptake were not related to cannabidiol treatment but to spontaneous remission of symptoms, a placebo effect (expectancy bias) or a test–retest variability of the PET assessment. However, a most recent study comparing 33 CHR and 19 healthy control individuals receiving a single oral dose of 600 mg of cannabidiol revealed a partial normalization of altered activity in the striatum, medial temporal cortex, and midbrain by cannabidiol as assessed by functional MRI (11), which is very much in line with our data (Figure 2B). In the same patients, the acute cannabidiol intake also attenuated the increased activation in the left insula/parietal operculum observed in CHR patients treated with a placebo during a monetary incentive delay fMRI task (25).

Furthermore, 32 of the CHR patients included in the abovementioned acute studies were also assessed regarding their anxiety and stress responses to the Trier Social Stress Test (TSST) after taking 600 mg cannabidiol or placebo for 1 week (26). CHR individuals who received cannabidiol showed an intermediate response regarding cortisol and anxiety changes associated with experimental stress compared to 26 healthy participants and the CHR placebo group. Thus, cannabidiol seems beneficial to the altered neuroendocrine and psychological responses to acute stress in CHR patients.

Given the first evidence of cannabidiol’s beneficial effects in CHR and first episode psychosis (FEP) and its excellent side-effect profile (8), cannabidiol may represent an interesting psychopharmacological approach with a unique mechanism of action compared to the currently approved antipsychotics (27).

## Data availability statement

The datasets presented in this article are not readily available because the dataset is subject to restrictions to access individual patients’ data in accordance with German law. An anonymous data PET data set is not available. Requests to access the datasets should be directed to FML, markus.leweke@zi-mannheim.de.

## Ethics statement

Ethical approval was not provided for this study on human participants because it was considered an individual treatment attempt that was reported to the ethics committee of the University of Cologne, which accepted the approach unconditionally. A formal approval could not be provided in accordance with German law. The patients/participants provided their written informed consent to participate in this study. Written informed consent was obtained from the individual(s) for the publication of any potentially identifiable images or data included in this article.

## Author contributions

DK and FML treated the patient and wrote the paper. LK and CR were involved in the PET investigations and analyzed corresponding data. CR critically contributed to manuscript preparation. All authors contributed to the article and approved the submitted version.

## Funding

The study received funding from the Stanley Medical Research Institute (01–315 and 03-NV-003 to FML), the Koeln Fortune Program (108–2000 to FML), and the BMBF (01KN0706 to DK). The funders were not involved in the study design, collection, analysis, interpretation of data, the writing of this article, or the decision to submit it for publication.

## Conflict of interest

FML and DK are shareholders of curantis UG (Ltd.). CR is a shareholder of lero bioscience UG (Ltd.) and is currently employed by Endosane Pharmaceuticals GmbH. FML received a research grant from Endosane Pharmaceuticals GmbH.

The remaining authors declare that the research was conducted in the absence of any commercial or financial relationships that could be construed as a potential conflict of interest.

## Publisher’s note

All claims expressed in this article are solely those of the authors and do not necessarily represent those of their affiliated organizations, or those of the publisher, the editors and the reviewers. Any product that may be evaluated in this article, or claim that may be made by its manufacturer, is not guaranteed or endorsed by the publisher.
